# Concordance between crude extract and component allergens in a multiple allergen simultaneous test: a large-scale retrospective analysis

**DOI:** 10.3389/bjbs.2026.16596

**Published:** 2026-07-16

**Authors:** Hyejin Ryu, Kuenyoul Park, Kina Kim, Dohsik Minn

**Affiliations:** 1 Department of Laboratory Medicine, Seegene Medical Foundation, Seoul, Republic of Korea; 2 Department of Laboratory Medicine, Hanyang University College of Medicine, Seoul, Republic of Korea

**Keywords:** allergy testing, component-resolved diagnostics, diagnostic concordance, multiplex immunoassay, specific IgE

## Abstract

**Objectives:**

Multiple allergen simultaneous tests (MAST) increasingly incorporate both crude extract and molecular component allergens for specific IgE testing. However, systematic data on the diagnostic concordance between these two approaches remain limited. We evaluated the agreement between crude extract and corresponding component allergen results using a MAST panel in a large clinical laboratory cohort.

**Methods:**

We retrospectively analysed deduplicated MAST results from a high-volume referral laboratory over a two-month period. For 29 crude extract allergens paired with 48 corresponding component allergens, positivity rates were assessed using a cutoff of class 2 or higher (≥0.70 IU/mL). Concordance was defined as both crude extract and at least one component allergen being positive, or both being negative. Agreement was evaluated using percent agreement and Cohen’s kappa coefficient.

**Results:**

A total of 19,949 patients were included. The highest positivity rates were observed for *Dermatophagoides farinae* (31.8%), *D. pteronyssinus* (29.9%), rDer f 2 (24.2%), and cat epithelium (12.2%). Overall percent agreement exceeded 90% for most allergen pairs, primarily driven by high negative agreement. Cohen’s kappa revealed substantial agreement (κ ≥ 0.6) for ten allergens including *D. farinae*, cat dander, and birch pollen, whereas eight allergens including *D. pteronyssinus* and milk showed poor agreement (κ < 0.1) due to low component allergen positivity despite positive crude extract results.

**Conclusion:**

Diagnostic concordance between crude extract and component allergen testing in MAST varies substantially across allergen sources. These findings underscore the need for allergen-specific interpretation guidelines when reporting MAST results incorporating component allergens in clinical laboratories.

## Introduction

Allergic diseases, with their continuously increasing global prevalence, manifest in diverse clinical presentations including respiratory allergies, food allergies, and anaphylaxis [1, 2], significantly impairing patients’ quality of life and potentially becoming life-threatening in severe cases [3–5]. Identification of causative allergens forms the foundation for establishing personalised treatment plans, including avoidance therapy, immunotherapy, and pharmacotherapy, thereby underscoring the critical importance of diagnostic testing for allergen identification [6].

Tests for confirming allergen sensitisation can be broadly categorised into *in vivo* and *in vitro* methods. *In vivo* tests include the skin prick test (SPT) and end-organ challenge tests, with oral food challenge (OFC) considered the gold standard for food allergy diagnosis [7]. SPT has been widely utilised as a screening test to confirm specific IgE (sIgE) sensitisation, offering advantages of low cost and rapid results. However, it has limitations including susceptibility to interference from antihistamine use, inability to perform in patients with skin conditions, and inter-observer variability in result interpretation [8].

To overcome these limitations, *in vitro* tests using serum specimens have been developed. Total IgE can be utilised for initial screening of allergic diseases [9], while sIgE tests are used to confirm sensitisation to specific allergens. Notably, the fluorescence enzyme immunoassay-based ImmunoCAP (Thermo Fisher Scientific, Phadia AB, Uppsala, Sweden) has become widely used as a singleplex sIgE test. Although ImmunoCAP provides safer and more standardised results compared to *in vivo* tests, it measures only one allergen at a time, requiring larger sample volumes, increased costs, and longer turnaround times when multiple allergens need to be assessed [10]. To address these drawbacks, multiple allergen simultaneous test (MAST) has been widely adopted [11]. MAST enables simultaneous measurement of sIgE against multiple allergens using small serum volumes, making it useful for screening allergen sensitisation [12].

However, conventional MAST panels primarily utilise crude allergen extracts, making it difficult to distinguish true sensitisation from cross-reactivity to other allergens, potentially resulting in false-positive or false-negative results [13]. Component-resolved diagnostics (CRD), which measures sIgE to individual allergen proteins rather than whole crude extracts, can help overcome these limitations by characterizing sensitisation profiles at the molecular level [14, 15]. While CRD has long been available on singleplex platforms such as ImmunoCAP, its incorporation into multiplex panels is a recent development. PROTIA Allergy-Q 192D (PROTIA 192D; ProteomeTech Inc., Seoul, Korea), approved by the Korean Ministry of Food and Drug Safety in 2024, is an enzyme immunoassay-based multiplex panel that simultaneously measures sIgE against 29 crude extract allergens and 48 component allergens within a single test [16]. Similarly, the ALEX 2 macroarray (Macro Array Diagnostics, Vienna, Austria) includes both allergen extracts and molecular components in one panel [17].

Although these platforms uniquely enable direct comparison of crude extract and component allergen results, discordant results between the two for the same allergen source may occur in clinical practice, potentially causing confusion in result interpretation. Previous studies on multiplex CRD platforms such as ALEX2 have primarily focused on inter-platform comparisons of component results [18, 19] rather than systematically analysing the concordance between crude extract and component allergen results within a single panel. Consequently, how clinicians should interpret discordant results between extract-based and component-based sIgE measured simultaneously remains largely unexplored.

In Korea, MAST is covered by national health insurance for patients with allergic diseases such as asthma, atopic dermatitis, allergic rhinitis, and anaphylaxis, as well as skin conditions including urticaria and contact dermatitis. Consequently, test data from large-scale referral laboratories represent valuable cohort data reflecting the characteristics of allergic patient populations nationwide.

In this study, we evaluated the concordance between crude extract allergens and their corresponding component allergens in PROTIA 192D using data from a high-volume referral laboratory. By characterizing the concordance and discordance patterns, we aimed to provide baseline data that can aid clinicians in interpreting positive results when both crude and component allergen tests are reported together.

## Materials and methods

### Study participants

This study analysed MAST test results extracted from the laboratory information system of a high-volume referral laboratory in Seoul, Korea, offering approximately 4,500 test items with a daily testing volume of 400,000 tests. Test results from August to September 2025 were included. During data extraction, duplicate tests from the same patient were identified based on patient name, patient identification number assigned by the referring institution, age and specimen collection office; in cases of repeated measurements, only the first reported result was included. All personal identifiers including patient name, institutional patient identifier, and date of birth were subsequently removed and replaced with study numbers for de-identification. A total of 19,949 test results were included in the final analysis. Patient demographics including age, sex, and institution type were extracted from patient records, and geographic region was classified based on the location of the specimen collection office. This study was conducted in accordance with the Declaration of Helsinki (as revised in 2013) and approved by the Institutional Review Board of Seegene Medical Foundation (SMF-IRB-2025-015). The requirement for informed consent was waived owing to the retrospective nature of the study.

### *In vitro* allergen sIgE measurements

PROTIA 192D testing was performed according to the manufacturer’s instructions. This multiplex immunoassay platform simultaneously measures total IgE, sIgE against crude extract allergens, and sIgE against component allergens. Results were reported as quantitative values (IU/mL) and semi-quantitative classes (class 0–6): class 0, negative (≤0.34 IU/mL); class 1, 0.35–0.69 IU/mL; class 2, 0.70–3.49 IU/mL; class 3, 3.50–17.49 IU/mL; class 4, 17.50–49.99 IU/mL; class 5, 50–99.99 IU/mL; class 6, ≥100 IU/mL. Positivity was defined as class ≥2 (≥0.70 IU/mL), consistent with the threshold applied in previous MAST studies [12, 16, 20]. Throughout this study, ‘positivity’ and sIgE results denote laboratory measurements.

### Statistical analysis

Continuous variables were presented as median with interquartile range (IQR), and categorical variables as frequencies with percentages. Positive rates for each crude extract allergen and its corresponding component allergens were visualised using bar charts. Inhalant and food allergens were presented separately. To evaluate concordance between crude extract allergens and their corresponding component allergens, overall percent agreement, positive percent agreement (PPA), negative percent agreement (NPA), and Cohen’s kappa coefficient were calculated. For PPA and NPA calculations, the crude extract allergen result served as the reference method, and the component result was considered positive if any corresponding component allergen showed positivity. Heatmaps were restricted to samples with positive crude extract results (class ≥2), as the overwhelming proportion of concordant negative pairs would obscure meaningful class distributions. This restriction entailed minimal information loss, given that isolated component positivity without crude extract positivity was rare (NPA >99% for most pairs). Descriptive statistics were calculated using R version 4.5.3 (R Foundation for Statistical Computing, Vienna, Austria) with tidyverse packages for data manipulation. Bar charts and heatmaps were generated using Python 3.12.12 with matplotlib. Concordance was assessed using percent agreement and Cohen’s kappa coefficient.

## Results

### Characteristics of study participants

A total of 19,949 subjects were included in this study (Table 1). The median age was 33 years (Q1–Q3: 14–53 years), with 28.6% aged <18 years, 30.1% aged 18–39 years, 28.7% aged 40–64 years, and 12.6% aged ≥65 years. Females comprised 54.4% of the study population. The majority of tests (83.7%) were requested from outpatient clinics. Geographically, as the referral laboratory is located in Seoul, specimens were predominantly collected from the Seoul Metropolitan Area: Gyeonggi (40.6%), Seoul (29.3%), and Incheon (19.1%), with 89.0% of subjects probably residing in the metropolitan region.

**TABLE 1:** Demographic and clinical characteristics of study participants.

Characteristic	Value
Total patients, N	19,949
Age (years), median (Q1–Q3)	33 (14–53)
Age group, N (%)	
<18 years	5,696 (28.6%)
18–39 years	6,018 (30.1%)
40–64 years	5,722 (28.7%)
≥65 years	2,513 (12.6%)
Female, N (%)	10,855 (54.4%)
Institution type, N (%)	
Clinic	16,697 (83.7%)
Hospital	2,927 (14.7%)
Screening center	192 (1%)
Unknown	133 (0.7%)
Region, N (%)	
Gyeonggi	8,095 (40.6%)
Seoul	5,848 (29.3%)
Incheon	3,807 (19.1%)
Gangwon	1,248 (6.3%)
Jeju	630 (3.2%)
Chungnam	158 (0.8%)
Jeonbuk	60 (0.3%)
Gwangju	49 (0.2%)
Chungbuk	36 (0.2%)
Busan	7 (0%)
Gyeongbuk	6 (0%)
Daegu	4 (0%)
Unknown	1 (0%)

### Positivity rates of crude extract and component allergens

**FIGURE 1:**
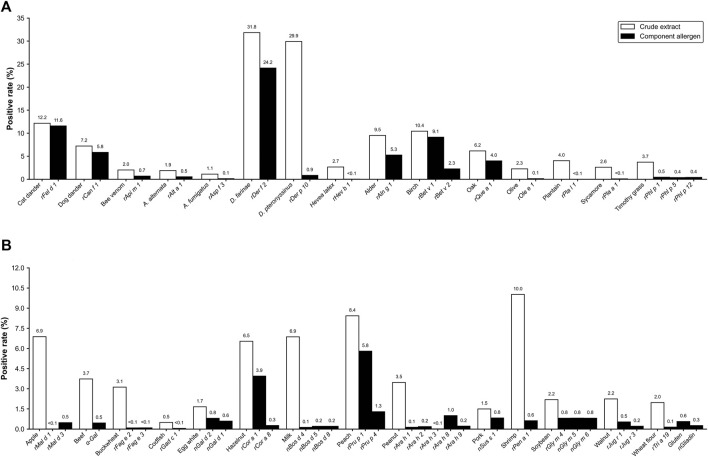
Positive rates of crude extract allergens and component allergens measured by PROTIA Allergy-Q 192D (N = 19,949). **(A)** Inhalant allergens and **(B)** food allergens are shown. Open bars indicate crude extract allergens and filled bars indicate component allergens. Numbers above bars indicate positive rates (%). Values less than 0.1% are shown as ”<0.1”.

Positivity rates were analysed for 29 crude extract allergens and 48 component allergens (Figure 1; Supplementary Table S1). Among crude extract allergens, the highest positivity rate was observed for *Dermatophagoides farinae* crude extract (31.8%), followed by *D. pteronyssinus* (29.9%). Other crude extracts with positivity rates ≥10% included cat epithelium and dander (12.2%), birch pollen (10.4%), and shrimp (10.0%), while most others showed positivity rates below 10%. Among component allergens, rDer f 2, the major component allergen of house dust mite, showed the highest positivity rate (24.2%), followed by rFel d 1 from cat epithelium and dander (11.6%) and rBet v 1 from birch pollen (9.1%).

Notable discrepancies between crude extract and component allergen positivity rates were observed for several allergens. For *D. pteronyssinus*, the crude extract positivity rate was 29.9%, whereas the corresponding component allergen rDer p 10 showed a positivity rate of only 0.9%. Similarly, shrimp crude extract had a positivity rate of 10.0%, but the corresponding component allergen rPen a 1 was positive in only 0.6% of cases. Comparable patterns were observed for apple and milk, where crude extract positivity rates were 6.9% for each, but all corresponding component allergens showed positivity rates below 1%.

### Concordance between crude extract and component allergen

Overall percent agreement between crude extract and corresponding component allergen tests exceeded 90% for most allergens (Table 2). Food allergens including codfish, egg white, and pork showed overall agreement rates above 99%, primarily driven by high NPA, indicating that cases with negative crude extract but positive component allergen results were rare. In contrast, PPA was below 90% for most allergens except birch (92.1%), and *D. pteronyssinus* showed the lowest overall agreement rate of 70.9% among all allergens.

**TABLE 2:** Concordance between crude extract allergens and component allergens in PROTIA 192D (N = 19,949).

Allergen group	Crude extract allergen	Component allergen	Crude extract allergen/Component allergen				Agreement (%)			Cohen’s kappa
			+/+	+/−	−/+	−/−	Positive	Negative	Overall	
Animals	Cat epithelium and dander	rFel d 1	2,159	267	152	17,371	89.0	99.1	97.9	0.9
​	Dog dander	rCan f 1	1,014	425	152	18,358	70.5	99.2	97.1	0.763
Foods	Apple	rMal d 1, rMal d 3	65	1,308	33	18,543	4.7	99.8	93.3	0.08
​	Beef	α-Gal	55	689	36	19,169	7.4	99.8	96.4	0.125
​	Buckwheat	rFag e 2, rFag e 3	22	601	10	19,316	3.5	99.9	96.9	0.064
​	Codfish	rGad c 1	10	88	0	19,851	10.2	100.0	99.6	0.184
​	Egg white	nGal d 1, nGal d 2	176	155	9	19,609	53.2	100.0	99.2	0.678
​	Hazelnut	rCor a 1, rCor a 8	764	540	56	18,589	58.6	99.7	97.0	0.704
​	Milk	nBos d 4, nBos d 5, nBos d 8	63	1,307	2	18,577	4.6	100.0	93.4	0.082
​	Peach	rPru p 1, rPru p 4	1,134	548	105	18,162	67.4	99.4	96.7	0.759
​	Peanut	rAra h 1, rAra h 2, rAra h 3, rAra h 8, rAra h 9	119	574	162	19,094	17.2	99.2	96.3	0.229
​	Pork	nSus s 1	141	158	24	19,626	47.2	99.9	99.1	0.604
​	Shrimp	rPen a 1	122	1,877	2	17,948	6.1	100.0	90.6	0.104
​	Soybean	rGly m 4, nGly m 5, nGly m 6	221	216	124	19,388	50.6	99.4	98.3	0.557
​	Walnut	rJug r 1, rJug r 3	140	308	6	19,495	31.2	100.0	98.4	0.465
​	Wheat flour	rTri a 19, Gluten, nGliadin	125	269	1	19,554	31.7	100.0	98.6	0.476
Insects	Bee venom	rApi m 1	139	263	1	19,546	34.6	100.0	98.7	0.508
Microorganisms	*Alternaria alternata*	rAlt a 1	82	294	24	19,549	21.8	99.9	98.4	0.335
​	*Aspergillus fumigatus*	rAsp f 3	15	207	10	19,717	6.8	99.9	98.9	0.119
Mites	*Dermatophagoides farinae*	rDer f 2	4,756	1,597	62	13,534	74.9	99.5	91.7	0.795
​	*D. pteronyssinus*	rDer p 10	161	5,806	9	13,973	2.7	99.9	70.9	0.037
Occupational	Hevea latex	rHev b 1	4	527	1	19,417	0.8	100.0	97.4	0.014
Pollens	Alder	rAln g 1	1,050	849	0	18,050	55.3	100.0	95.7	0.691
​	Birch	rBet v 1, rBet v 2	1,919	164	58	17,808	92.1	99.7	98.9	0.939
​	Oak	rQue a 1	735	494	61	18,659	59.8	99.7	97.2	0.712
​	Olive	rOle e 1	18	433	10	19,488	4.0	99.9	97.8	0.073
​	Plantain	rPla l 1	1	802	0	19,146	0.1	100.0	96.0	0.002
​	Sycamore	rPla a 1	15	505	4	19,425	2.9	100.0	97.4	0.054
​	Timothy grass	rPhl p 1, rPhl p 5, rPhl p 12	184	556	17	19,192	24.9	99.9	97.1	0.381

**FIGURE 2:**
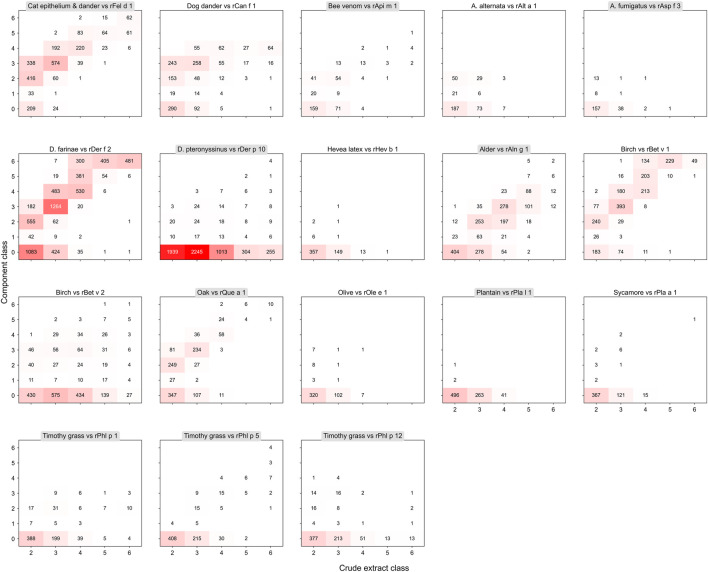
Concordance patterns between crude extract and component allergens for inhalant allergens. Heatmaps show the distribution of component allergen classes according to crude extract allergen classes. Only samples with positive crude extract results (class ≥2) are shown. Numbers within cells indicate the number of samples. Color intensity reflects sample count (white to red).

**FIGURE 3:**
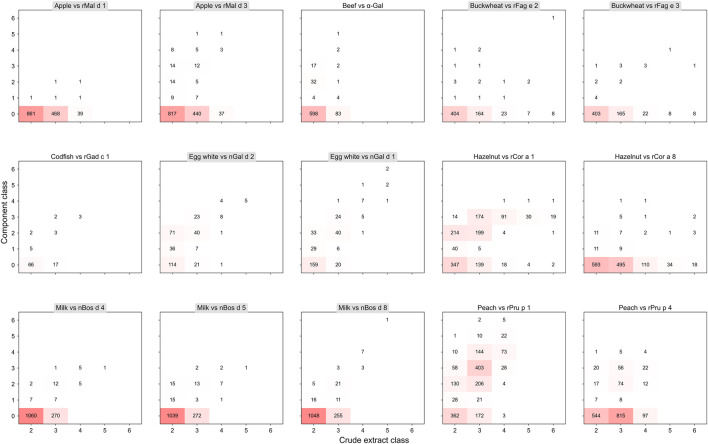
Concordance patterns between crude extract and component allergens for food allergens (Apple–Peach). Heatmaps show the distribution of component allergen classes according to crude extract allergen classes. Only samples with positive crude extract results (class ≥2) are shown. Numbers within cells indicate the number of samples. Color intensity reflects sample count (white to red).

**FIGURE 4:**
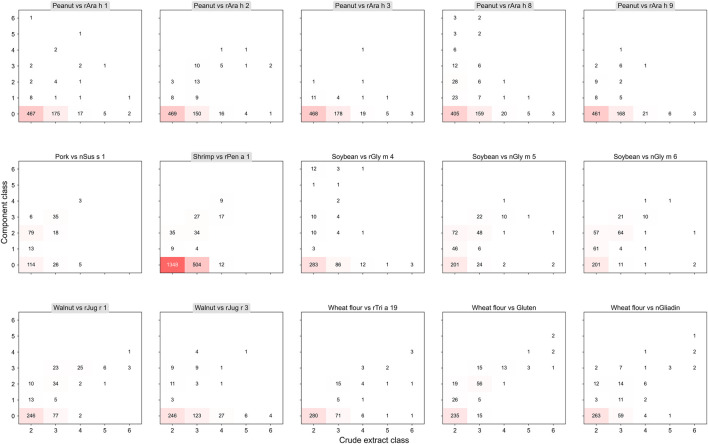
Concordance patterns between crude extract and component allergens for food allergens (Peanut–Wheat flour). Heatmaps show the distribution of component allergen classes according to crude extract allergen classes. Only samples with positive crude extract results (class ≥2) are shown. Numbers within cells indicate the number of samples. Color intensity reflects sample count (white to red).

Concordance was further evaluated using Cohen’s kappa coefficient (Table 2) and visualised with heatmaps (Figures 2–4). Birch (κ = 0.939) and cat dander (κ = 0.900) demonstrated almost perfect agreement (κ ≥ 0.8), while dog dander, D. farinae, peach, oak, hazelnut, alder, egg white, and pork showed substantial agreement (κ ≥ 0.6). For these allergen pairs with high concordance, heatmap analysis demonstrated similar class distributions between crude extract and component allergen results. Conversely, eight allergens including apple, milk, olive, buckwheat, sycamore, *D. pteronyssinus*, Hevea latex, and plantain showed very low agreement (κ < 0.1). For example, among patients with positive crude extract results for *D. pteronyssinus*, more than 90% showed negative results for the component allergen test, indicating that the causative component allergen could not be identified using this panel.

## Discussion

This study is a large-scale investigation analysing the positivity rates and concordance between crude extract allergens and component allergens using the PROTIA 192D panel in 19,949 patients with suspected allergic diseases in Korea. The median age of study subjects was 33 years, with females comprising 54.4%, which is consistent with previous reports that the prevalence and healthcare utilisation for allergic diseases are higher in adult women [21, 22]. Overall percent agreement exceeded 90% for most allergen pairs; however, concordance varied substantially across allergen sources, with Cohen’s kappa ranging from almost perfect agreement for birch (κ = 0.939) and cat dander (κ = 0.900) to very low agreement (κ < 0.1) for eight allergens including *D. pteronyssinus*, apple, and milk. To our knowledge, this is the first study to systematically analyse extract–component concordance across a broad range of allergen sources within a single multiplex panel, and the 29 crude extract–48 component allergen pair analysis presented here provides a comprehensive reference for interpreting these paired results in clinical practice.

The allergens with the highest positivity rates in this study were house dust mites, with *D. farinae* at 31.8% and *D. pteronyssinus* at 29.9%. This is consistent with previous reports that 40%–60% of Korean patients with respiratory allergies are sensitised to house dust mites and that house dust mites are detected in more than 90% of Korean households [23]. A nationwide multicenter study also reported house dust mites as the most common sensitising allergen [24]. Among food allergens, shrimp showed the highest positivity rate at 10.0%, followed by milk at 6.9%, apple at 6.9%, and peanut at 3.5%. This high positivity rate to crustaceans is consistent with previous studies reporting seafood as the most common cause of food allergy in Korean adults and reflects the epidemiological characteristics of high shrimp and crab allergy prevalence in Asian regions [25]. Among pollen allergens, birch was highest at 10.4%, which is similar to the 8%–16% birch pollen sensitisation rate reported in the European general population [26]. For component allergens, rDer f 2, a major allergen of *D. farinae*, showed the highest positivity rate at 24.2%, followed by rFel d 1 at 11.6% and rBet v 1 at 9.1%.

The analysis revealed that overall agreement between crude extract allergens and component allergens was high at over 90% for most allergens; however, this was primarily due to the majority being concordant negative results, and positive agreement varied greatly depending on the allergen source. Because overall agreement is dominated by concordant negative pairs, it can mask substantial discordance among sIgE-positive cases; positive percent agreement and Cohen’s kappa therefore provide a more informative measure of extract–component concordance than overall agreement alone. NPA was very high at over 99% for most crude extract–component allergen pairs, suggesting that component allergen testing is highly useful for ruling out sensitisation to specific allergens. Additionally, the high NPA indicates that crude extract testing adequately captures most sensitised patients, and isolated component allergen positivity without crude extract reactivity is uncommon. However, PPA showed a wide distribution ranging from 0.1% to 92.1% depending on the allergen, indicating that the proportion of patients positive for the corresponding component among crude extract–positive patients varied greatly by allergen. For example, plantain, which showed the lowest PPA, demonstrated that the two tests provide different information, warranting caution in clinical interpretation. Such discordance suggests that a structured approach considering the overall context, not just single positive or negative results, is necessary when interpreting CRD results [27].

The discordance between crude extract and component allergens can be explained by several factors. First, cross-reactive carbohydrate determinants (CCDs) are N-glycan structures present in plant- and insect-derived allergens that can cause false-positive reactions in up to 30% of patients but do not induce clinically significant allergic symptoms [28, 29]. Second, IgE to pan-allergens such as profilin can result in positive crude extract allergen results but negative component allergen results [30]. Third, this discrepancy may reflect insufficient inclusion of major components of the allergen source in the current multiplex panel, or cases like shrimp or milk where causative component allergens are diverse and difficult to include comprehensively in a single panel [31, 32]. In such cases, discordant results may reflect either false-positive crude extract reactions due to CCDs or pan-allergens, or false-negative component results due to incomplete coverage of clinically relevant components in the panel. Therefore, clinicians should interpret discordant results in the context of clinical synopsis and, where indicated, consider confirmatory testing with singleplex assays.

For house dust mites, rDer f 2, a major allergen of *D. farinae*, showed a positivity rate of 24.2% with relatively high concordance with crude extract, because Der f 2 is a major allergen that induces IgE responses in more than 70% of patients [33]. However, the positivity rate of rDer p 10, a component allergen of *D. pteronyssinus*, was only 0.9%. This discordance occurs because Der p 10, as a tropomyosin, is a minor allergen of house dust mites showing IgE reactivity in only about 7%–15% of allergic patients; however, it is included in the panel because it is well known as a cross-reactive allergen in invertebrates, and this point should be noted when interpreting test results [33, 34]. Therefore, when Der p 10 is positive in the laboratory, it may be advisable to include a comment noting that this allergen is a well-known cross-reactive allergen.

Birch and rBet v 1 showed positivity rates of 10.4% and 9.1%, respectively, with Cohen’s kappa of 0.939 demonstrating excellent agreement between the two tests. Bet v 1 is a major allergen that induces IgE responses in more than 90% of birch pollen allergy patients [35], and this study confirmed its reliability as a marker allergen through high concordance with crude extract. The alder/rAln g 1 and oak/rQue a 1 pairs also showed relatively high concordance with PPA of 55.3% and 59.8%, respectively. Since these allergens all belong to the PR-10 family and share high homology with Bet v 1 [36], they demonstrate good concordance between component allergens and crude extracts in tree pollen allergy diagnosis.

Shrimp crude extract showed the highest positivity rate (10.0%) among food allergens; however, the positivity rate of rPen a 1 (tropomyosin) was only 0.6%, resulting in very low PPA of 6.1%. This finding may be attributed to shrimp sensitisation being mediated by various allergens beyond tropomyosin, including arginine kinase and sarcoplasmic calcium-binding protein [31, 37]. Therefore, simply interpreting positive shrimp crude extract results as tropomyosin sensitisation is inappropriate, and this suggests that comprehensive evaluation of various shrimp allergen components beyond tropomyosin is necessary for CRD.

Meanwhile, among food allergens, some items including milk, apple, and peanut showed low concordance between crude extract and component allergens. The crude extract positivity rate for milk was 6.9%, but the positivity rates for nBos d 4 (α-lactalbumin), nBos d 5 (β-lactoglobulin), and nBos d 8 (casein) were remarkably low at 0.2%, 0.1%, and 0.2%, respectively. This likely reflects the highly diverse IgE responses to milk proteins with large individual variation, making it difficult to represent with specific components [38]. Apple also showed low positivity rates of 0.0% and 0.5% for rMal d 1 and rMal d 3, respectively, compared to the crude extract positivity rate of 6.9%. One possible explanation is clinically irrelevant positive reactions caused by pan-allergens such as CCDs or profilin contained in the extract, or sensitisation to other allergens not included in the panel such as Mal d 2 and Mal d 4 [39]. For peanut, rAra h 8 (PR-10 protein) showed the highest positivity rate at 1.0% compared to the crude extract positivity rate of 3.5%, and since it shares high homology with Bet v 1 [40], the possibility of cross-reactivity with birch pollen cannot be excluded considering the high birch positivity rate of 10.4% in this study. In contrast, peach, hazelnut, and egg white showed substantial agreement (κ ≥ 0.6), suggesting that the utility of CRD in food allergens should be evaluated differently according to the characteristics of individual allergens.

Integrating component allergens into multiplex testing platforms provides significant clinical advantages. The previously developed PROTIA Allergy-Q 64 Atopy (ProteomeTech Inc., Seoul, Korea), which included a limited set of 10 component allergens (Der f 1/2, Bet v 1, Fel d 1, Que a 1, α-lactalbumin, β-lactoglobulin, casein, ω-5 gliadin, α-Gal), showed high concordance (≥88%) and correlation (r > 0.640) compared to ImmunoCAP, with particularly high correlations for α-lactalbumin (r = 0.963), ω-5 gliadin (r = 0.931), and Bet v 1 (r = 0.855), confirming high accuracy for component allergens in such multiplex systems [41]. The PROTIA 192D used in this study is an expanded multiplex system that can simultaneously measure 48 component allergens, enabling simultaneous testing of a broad allergen panel in a time- and cost-efficient manner, and can distinguish primary sensitisation from cross-reactivity. For example, patients positive for both peanut extract and Ara h 2 are at high risk for systemic reactions, whereas patients positive only for Ara h 8 are likely to experience only mild oral allergy syndrome [40]. Such risk stratification provides important information for patient management.

This study has several limitations. First, due to the retrospective study design, clinical confirmatory tests such as SPT or OFC tests were not performed, making it difficult to directly evaluate the relationship between serological sensitisation and clinical allergy, or to determine whether discordant results arose from false-positive crude extract or false-negative component reactions. Second, as the majority of specimens were collected from the Seoul Metropolitan Area, the findings may not be fully generalisable to the entire Korean population; moreover, the allergen positivity profile reflects Korean and East Asian exposure patterns, so the concordance estimates may differ in other populations or when other multiplex platforms with different panel compositions are used. Third, due to limitations of the component allergen panel included in the PROTIA 192D system, some major component allergens (e.g., Der p 1, Der p 23, Ara h 6) were not included in the analysis, which may partly account for the low extract–component concordance observed for certain sources; inclusion of these component allergens could be considered when developing new panels in the future. Fourth, concordance was assessed at a single positivity threshold; a lower or higher cutoff would alter positive rates and could shift the positive percent agreement, so the concordance estimates should be interpreted with reference to the threshold used.

In conclusion, this study is the first to systematically compare sIgE positivity patterns between crude extract allergens and component allergens using the PROTIA 192D panel, confirming that positivity rates and concordance differ depending on the allergen. High concordance was observed for birch and *D. farinae*, where component allergens greatly assist in interpreting crude extract results, while low concordance was observed for shrimp and milk, requiring integrated interpretation of both test results. The results of this study emphasise the importance of customised interpretation considering allergen-specific characteristics in the clinical application of CRD, and further studies linking clinical information, such as symptom history or challenge test results, will be needed in the future.

This work represents an advance in biomedical science because it provides the first systematic comparison of crude extract and component allergen concordance in a 192-allergen multiplex panel.

## Summary table

### What is known about this subject

- Multiplex allergen tests now include both crude extract and molecular component allergens for specific IgE measurement.
- Component-resolved diagnostics can improve specificity by distinguishing genuine sensitisation from cross-reactivity.
- Discordant results between crude extract and component allergens may occur but systematic data remain limited.

### What this paper adds

- Overall agreement exceeded 90% for most allergen pairs but positive agreement varied widely across allergen sources.
- House dust mite, birch, and cat allergens showed high concordance while shrimp, milk, and apple showed poor concordance.
- Allergen-specific interpretation guidelines are needed when reporting multiplex tests incorporating component allergens.

## Data Availability

The datasets presented in this article are not readily available because the individual-level data cannot be shared publicly due to Institutional Review Board restrictions and patient privacy protection policies. Requests to access the datasets should be directed to Hyejin Ryu, hjryu@mf.seegene.com.
