# Supplementary material for: Concordance between crude extract and component allergens in a multiple allergen simultaneous test: a large-scale retrospective analysis
Source: Br J Biomed Sci. 2026 Jul 16;83:16596. doi: 10.3389/bjbs.2026.16596 (PMC13422201; doi:10.3389/bjbs.2026.16596)
Supplement: Supplementary file 1 [file Table1.DOCX]

**Supplementary Table 1**. Positivity rates of crude extract allergens and their corresponding component allergens measured by the PROTIA Allergy-Q 192D MAST (N = 19,949)

| **Allergen group** | **Crude extract allergen** | **Component allergen** | **Crude extract positivity, n (%)** | **Component allergen positivity, n (%)** |
| --- | --- | --- | --- | --- |
| Animals | Cat epithelium & dander | rFel d 1 | 2,426 (12.2) | 2,311 (11.6) |
|  | Dog dander | rCan f 1 | 1,439 (7.2) | 1,166 (5.8) |
| Foods | Apple | rMal d 1 | 1,373 (6.9) | 2 (<0.1) |
|  |  | rMal d 3 |  | 96 (0.5) |
|  | Beef | α-Gal | 744 (3.7) | 91 (0.5) |
|  | Buckwheat | rFag e 2 | 623 (3.1) | 18 (0.1) |
|  |  | rFag e 3 |  | 19 (0.1) |
|  | Codfish | rGad c 1 | 98 (0.5) | 10 (0.1) |
|  | Egg white | nGal d 2 | 331 (1.7) | 160 (0.8) |
|  |  | nGal d 1 |  | 118 (0.6) |
|  | Hazelnut | rCor a 1 | 1,304 (6.5) | 787 (3.9) |
|  |  | rCor a 8 |  | 53 (0.3) |
|  | Milk | nBos d 4 | 1,370 (6.9) | 26 (0.1) |
|  |  | nBos d 5 |  | 41 (0.2) |
|  |  | nBos d 8 |  | 41 (0.2) |
|  | Peach | rPru p 1 | 1,682 (8.4) | 1,157 (5.8) |
|  |  | rPru p 4 |  | 258 (1.3) |
|  | Peanut | rAra h 1 | 693 (3.5) | 27 (0.1) |
|  |  | rAra h 2 |  | 37 (0.2) |
|  |  | rAra h 3 |  | 3 (<0.1) |
|  |  | rAra h 8 |  | 200 (1.0) |
|  |  | rAra h 9 |  | 44 (0.2) |
|  | Pork | nSus s 1 | 299 (1.5) | 165 (0.8) |
|  | Shrimp | rPen a 1 | 1,999 (10.0) | 124 (0.6) |
|  | Soybean | rGly m 4 | 437 (2.2) | 162 (0.8) |
|  |  | nGly m 5 |  | 163 (0.8) |
|  |  | nGly m 6 |  | 162 (0.8) |
|  | Walnut | rJug r 1 | 448 (2.2) | 107 (0.5) |
|  |  | rJug r 3 |  | 43 (0.2) |
|  | Wheat flour | rTri a 19 | 394 (2.0) | 29 (0.1) |
|  |  | Gluten |  | 113 (0.6) |
|  |  | nGliadin |  | 52 (0.3) |
| Insects | Bee venom | rApi m 1 | 402 (2.0) | 140 (0.7) |
| Microorganisms | *Alternaria alternata* | rAlt a 1 | 376 (1.9) | 106 (0.5) |
|  | *Aspergillus fumigatus* | rAsp f 3 | 222 (1.1) | 25 (0.1) |
| Mites | *Dermatophagoides farinae* | rDer f 2 | 6,353 (31.8) | 4,818 (24.2) |
|  | *D. pteronyssinus* | rDer p 10 | 5,967 (29.9) | 170 (0.9) |
| Occupational | Hevea latex | rHev b 1 | 531 (2.7) | 5 (<0.1) |
| Pollens | Alder | rAln g 1 | 1,899 (9.5) | 1,050 (5.3) |
|  | Birch | rBet v 1 | 2,083 (10.4) | 1,823 (9.1) |
|  |  | rBet v 2 |  | 451 (2.3) |
|  | Oak | rQue a 1 | 1,229 (6.2) | 796 (4.0) |
|  | Olive | rOle e 1 | 451 (2.3) | 28 (0.1) |
|  | Plantain | rPla l 1 | 803 (4.0) | 1 (<0.1) |
|  | Sycamore | rPla a 1 | 520 (2.6) | 19 (0.1) |
|  | Timothy grass | rPhl p 1 | 740 (3.7) | 90 (0.5) |
|  |  | rPhl p 5 |  | 76 (0.4) |
|  |  | rPhl p 12 |  | 81 (0.4) |

Abbreviations: MAST, multiple allergen simultaneous test; IgE, immunoglobulin E.

Positivity was defined as class ≥2 (specific IgE ≥0.70 IU/mL) using the PROTIA Allergy-Q 192D system (ProteomeTech Inc., Seoul, Korea).
